# Prevalence of iodine deficiency among vegan compared to vegetarian and omnivore children in the Czech Republic: cross-sectional study

**DOI:** 10.1038/s41430-023-01312-9

**Published:** 2023-07-24

**Authors:** Martin Světnička, Marina Heniková, Eliška Selinger, Anna Ouřadová, Jana Potočková, Tilman Kuhn, Jan Gojda, Eva El-Lababidi

**Affiliations:** 1 Centre for Research on Diabetes Metabolism, and Nutrition of Third Faculty of Medicine, Charles University, Prague, Czech Republic; 2 Department of Internal Medicine, University Hospital Královské Vinohrady and Third Faculty of Medicine, Charles University, Prague, Czech Republic; 3 Department of Paediatrics, University Hospital Královské Vinohrady and Third Faculty of Medicine, Charles University, Prague, Czech Republic; 4 The National Institute of Public Health, Centre for Public Health Promotion, Prague, Czech Republic; 5 The Institute for Global Food Security, Queen’s University Belfast, Belfast, Northern Ireland UK; 6 Heidelberg Institute of Global Health (HIGH), Faculty of Medicine and University Hospital, Heidelberg, Germany; 7 Department of Nutritional Sciences, University of Vienna, Vienna, Austria; 8 Medical University of Vienna, Centre for Public Health, Vienna, Austria

**Keywords:** Nutrition, Biomarkers, Malnutrition, Risk factors, Paediatrics

## Abstract

**Background:**

The intake and homeostasis of iodine, an essential micronutrient that plays a vital role in thyroid physiology, is of particular concern with the increasing popularity of vegetarian (VG) and vegan (VN) diets. Children on these restrictive diets may be at risk of possible adverse effects on growth and development, but there is currently a lack of recent epidemiological studies on this topic.

**Methods:**

We gathered clinical, anthropometric, and blood/urine data on iodine status and thyroid function from children aged 0–18 years who followed either a VG diet (*n* = 91), VN diet (*n* = 75), or omnivore diet (OM, *n* = 52). Cross-sectional comparison of the groups and linear regression was used. Stratified analyses were performed based on age (according to WHO): 0–5 years and 6–18 years.

**Results:**

Our study revealed no significant differences in levels of thyroid-stimulating hormone (TSH), triiodothyronine (fT3), thyroglobulin (TG) or anti-thyroid peroxidase antibody (ATPOc) between the VG, VN, and OM groups. However, thyroxine (fT4) levels were found to be higher in the VN group compared to the OM group (15.00 ± 1.73 vs. 16.17 ± 1.82 pmol/l, *p* < 0.001). The presence of anti-thyroglobulin antibodies (AhTGc) was notably more common in the VG (18.2%)/VN (35.0%) groups than in the OM group (2.1%) (*p* < 0.001). Regarding iodine status, the concentration of iodine in spot urine (UIC) was found to be highest in the OM group (197.28 ± 105.35 vs. VG: 177.95 ± 155.88 vs. VN: 162.97 ± 164.51 µg/l, *p* < 0.001). Notably, the lowest (5.99 µg/l) and highest (991.80 µg/l) levels were measured in the VN group. Of the participants, 31 VN, 31 VG and 10 OM children met the criteria for iodine deficiency (i.e., UIC < 100 µg/l). We found that children with regular iodine supplementation had higher UIC (*p* < 0.001). Importantly, the median UIC was above 100 µg/l in all three groups, through the recommended intake (RDI) of iodine was rarely met throughout the groups.

**Conclusion:**

We have observed a trend to lower UIC values in VN as compared to OM. This trend is also reflected in the median UIC values, even though the median UIC values were above the WHO cut-off (e.g., 100 µg/l) for iodine deficiency in all dietary groups. These results suggest that VN and VG children may be more at higher risk of iodine deficiency, this theory is also supported by higher prevalence of AhTGc positivity. Further research is needed to investigate the long-term impact of these dietary patterns on iodine status and thyroid function in children. Given our findings, it may also be necessary to consider new guidelines for supplementing children following VG and VN diets to ensure their iodine needs are met.

## Introduction

The increasing popularity of plant-based diets in recent years has been driven by various motives such as perceived health benefits, environmental concerns, and ethical considerations [1, 2]. These diets encompass a range of patterns with varying degrees of restriction, including lacto-ovo-vegetarian, ovo-vegetarian, lactovegetarian, and vegan or strict vegetarian diets, which exclude different types of animal products [3]. However, reducing the intake of animal foods may lead to inadequate nutrient intake in the long term [1]. Unfortunately, dietary guidelines from nutritional and pediatric societies regarding iodine intake among children following VG and VN diets are currently inadequate, apart from unserious online information sources, despite the growing number of families following these diets [4]. It is important to ensure that children following plant-based diets receive adequate amounts of all essential nutrients, including iodine, for their growth and development. Therefore, more research is needed to better understand the nutrient status of children following these dietetic patterns, as well as to develop appropriate dietary guidelines for this population.

Iodine, an essential micronutrient, is not evenly distributed in our environment, and insufficient soil contents in many areas of the world increase the risk of low iodine intake. The primary dietary sources of iodine are iodized table salt, dairy, eggs, and seafood [5]. Despite efforts to address this issue, the prevalence of iodine deficiency in European countries remains high at 53.8% for adults and 6.3% for children in general [6]. Currently, there is no available data on biomarkers of iodine status in the pediatric population consuming plant-based diets. In the German cross-sectional VeChi diet study, the VN group had the lowest iodine intake compared to the OM group; however, none of the examined groups met the RDI for iodine [7]. Our theoretical knowledge draws from studies on the adult population, which demonstrate that excluding all animal sources of iodine from the diet reduces the daily intake of iodine to 30 μg, compared to 110–130 μg in adults who consume a conventional diet including dairy, fish, and meat [8, 9]. The prevalence of iodine deficiency among adult VN living in Europe is higher than in OM [10]. Prolonged insufficient iodine intake may lead to iodine deficiency disorders (IDD’s). In pregnant women, iodine deficiency may lead to a disruption in the development of the fetal nervous system [11, 12]. The most severe form of iodine deficiency is endemic cretinism [11]. Mild iodine deficiency may result in developing endemic cognitive deficits, which can impede children from reaching their full cognitive potential [11, 12]. Children with severe iodine deficiency have been observed to experience a significant decrease in IQ and a higher prevalence of goiter [11, 12]. Conversely, long-term consumption of iodine exceeding the upper limit of the daily requirement could also lead to thyroid disease [13, 14]. In this study, we aimed to examine the nutritional intake and biomarkers of iodine status in children following VN and VG diets and compare them with OM children.

## Materials and methods

### Study design and participants

Between November 2019 and July 2021, a total of 222 children were recruited for the study. The participants were recruited through a network of general practitioners, social media, and vegan-focused web pages. Of the total participants, 92 were VG, 78 were VN, and 52 were OM who served as controls. Flow diagram of the study enrollment is depicted in Fig. S1. The inclusion criteria for participants were (1) self/parent-reported VG or VN or OM children (OM defined as consuming meat, dairy, and eggs on a regular basis, VG as not eating meat/meat products/fish but consuming dairy/eggs, and VN as not consuming any food of animal origin), and (2) age ranging from 0 to 18 years. Subjects with any chronic disease that could affect nutrient absorption (such as enteropathy, pancreatic insufficiency, etc.), metabolic diseases like phenylketonuria, or fructose malabsorption and children with autoimmune thyroid disease (AIT) were not enrolled in the study. The study was conducted at the Department of Paediatrics, Third Faculty of Medicine, Charles University, University Hospital Královské Vinohrady.

The study was conducted according to the Declaration of Helsinki guidelines and approved by the Ethics Committee of the Third faculty, Charles University, Prague, and Ethics Committee of Faculty Hospital Královské Vinohrady. All examinations were performed with parental written consent, with no financial incentives.

### Examination, medical history

Trained clinicians conducted structured medical interviews that focused on dietary habits and iodine intake. The participants were asked to self-identify as VN/VG/OM, and their dietary habits were examined accordingly. Specifically, the use of iodine supplements and their dosages (in µg), frequency of usage (e.g., daily, twice, or three times a week), and regularity (e.g., regular, irregular) were assessed through questionnaires. The parents of the participants were asked to fill in the questionnaires under medical supervision to ensure accuracy. Participants who did not take any supplements containing iodine were classified as “No” in supplement use, while those who did use supplements were classified as “Yes”.

### Laboratory analysis

Blood samples were collected from participants after an overnight fast through venepuncture, and spot urine samples were collected in the morning after an overnight fast. In cases where participants were infants or toddlers, blood samples were obtained after breastfeeding or a small breakfast. The biomarkers of interest included serum TSH, fT4, fT3, TG, and levels of antibodies, namely ATPOc and AhTGc, and UIC, the analyses were performed immediately in an ISO-certified institutional laboratory using validated routine methods. All parameters (TSH, fT4, fT3, ATPOc and AhTGc) were analyzed using chemiluminescence immunoassay automatically on the Siemens Atellica Solution system. For TG measurements, the electrochemiluminiscent elecsys TG assay on the Roche Cobas e411 analyzer was used. UIC was assessed using high-performance liquid chromatography. The reference values provided by the manufacturer were used for all parameters. Please see Table S1 for more details.

### Nutritional assessment

We used a 3-day weighed dietary record method to evaluate the iodine intake of our VG, VN, and OM participants. Parents were responsible for weighing and documenting all food and beverage items consumed by their children over three consecutive days (two weekdays and one weekend day) using electronic kitchen scales. In instances where accurate weighing was not possible (such as eating out), household measures (such as spoons, cups, and slices) and a photo booklet depicting child portion sizes of special VG and VN food were used to facilitate semi-quantitative recording. Any missing data were assessed by the study staff, who requested the necessary information from parents via electronic communication. Breast milk consumption was estimated based on maternal records and calculated using information that provides an estimate of the daily amount of milk a child could consume. The source of this information was the general recommendations for breast milk intake according to WHO and ESPHGAN [15]. We manually identified iodine sources from all the collected 3-day dietary records. Nutrient data, particularly iodine content, was obtained from the Czech database NutriDatabaze.cz, version 8.2, ÚZEI, Praha (http://www.nutridatabaze.cz/), which is part of EuroFIR (https://www.eurofir.org/food-information/food-composition-databases/). If a food source that contained iodine was not listed in the Czech database, we searched for iodine content in common foods in the USDA, FDA, and ODS-NIH database for iodine content in common foods Release 2.0 (2022; https://www.ars.usda.gov/northeast-area/beltsville-md-bhnrc/beltsville-human-nutrition-research-center/methods-and-application-of-food-composition-laboratory/mafcl-site-pages/iodine/). For products used solely in Europe, such as infant formula, which were not listed in the NutriDatabaze.cz, the dietitian registered the iodine content from the product packaging. We did not include iodine-containing supplements in this analysis of iodine intake. All the dietary data were calculated by a single dietitian. Please refer to Table S2.

### Anthropometrics

The participants' body height and body weight were measured three times using calibrated scales, and the average was calculated. The length of infants and toddlers was measured using an infantometer, while the heights of older children were measured with a stadiometer. The measurements were then transformed into percentiles using standard percentile graphs that have been validated for use in the Czech Republic (publicly accessible online: “6. National Anthropological Research 2001”) [16].

### Statistical analysis

Participants were stratified based on self-reporting into three dietary groups (VG, VN and OM). For VG and VN, participants were further classified according to their iodine supplementation habits as supplementing or not supplementing. New categorical variables were created classifying the participants as “deficient” or “not deficient” based on fulfillment of the laboratory criteria for iodine deficiency using various laboratory markers. For descriptive analyses, mean and standard deviations were calculated for numerical variables, with median and IQR calculated for variables with skewed distribution, while proportions and absolute numbers were reported for categorical variables. To evaluate the association of the diets with iodine status, effect sizes and their corresponding 95% confidence intervals, and *p* values were calculated to quantify differences between dietary and supplementation groups using the Wilcoxon signed-rank tests. Additionally, a simple linear regression model was employed to examine the relationship between log-transformed continuous values and diet type, with adjustments made for age and sex. For categorical variables, the chi-square test was used to calculate *p* values for differences between groups. To ensure the robustness of the findings, a sensitivity analysis was conducted by excluding participants diagnosed with AIT, as they displayed extreme values for TSH and antibody tests. The statistical analysis was done using RStudio software.

## Results

### Sample characteristics

The final sample consisted of *n* = 222 children, i.e., *n* = 92 VG, *n* = 78 VN and *n* = 52 OM. The mean age of participants was 5.4 years (±4.3 years) for VG, 4.4 years (±5.5 years) for VN and 6.7 years (±5.6 years) for OM and ranged from 0.5 to 18.5 years overall. The median age was 4.0 years for VG, 2.0 years for VN and 4.5 years for OM. There were no significant differences in age observed between the VG and VN groups when compared to the OM group. However, a significant difference was found between the VN and OM groups (*p* < 0.05). Due to this uneven distribution, we made the decision to divide our study group into two subgroups: “pre-schoolers,” encompassing children aged 0.5–6 years, and “older children and adolescents,” consisting of children aged 6–18.5 years. In the pre-schoolers group, there were a total of *n* = 28 participants in the OM group, *n* = 54 participants in the VG group, and *n* = 60 participants overall. As for the older children and adolescents group, there were *n* = 24 participants in the OM group, *n* = 38 participants in the VG group, and *n* = 18 participants in the VN group. There were no significant differences in sex, height percentile, weight percentile between the groups, but we observed a higher number *n* = 7 (e.g., 9%) of VN children with lower BMI i.e., below the 3rd percentile (*p* = 0.006). Please refer to Table 1.

**Table 1:** Description of the baseline characteristics of sample divided by dietary group VN/VG/OM.

Group	*n*	Sex		Median age	IQR	p**	Age range	
		Female	Male					
(a) Sex and age								
VN	78	38	40	2.0	1.0–3.94	0.002	0.5–18 years	
VG	92	54	38	4.0	2.0–8.0		0.5–18 years	
OM	52	25	27	4.5	2.0–10.94		0.75–18.5 years	

Variable		Omnivore		Vegetarian		Vegan		p*
		Median	IQR	Median	IQR	Median	IQR	
(b) Anthropometrics								
Height percentile		45.0	23.0–70.0	50.0	25.5–70.0	48.5	28.25–72.75	0.9
Weight percentile		44.0	16.0–65.5	46.5	19.75–70.0	40.0	13.0–64.0	0.6
BMI percentile		40.0	19.5–55:0	41.0	19.75–70.0	35.5	18.25–63.5	0.006

		*n* (total)		*n* (total)		*n* (total)		
Height ≤3 perc.		2		3		2		
Weight ≤3 perc.		5		5		6		
BMI ≤3 perc.		1		0		7		

*OM* omnivore, *VG* vegetarian, *VN* vegan, *n* number of subjects, *IQR* interquartile range, *perc.* percentile.

*p value calculated using Wilcoxon signed-rank tests; p** = *p* value for median age between all three groups.

### Iodine intake and supplementation

**Fig. 1:**
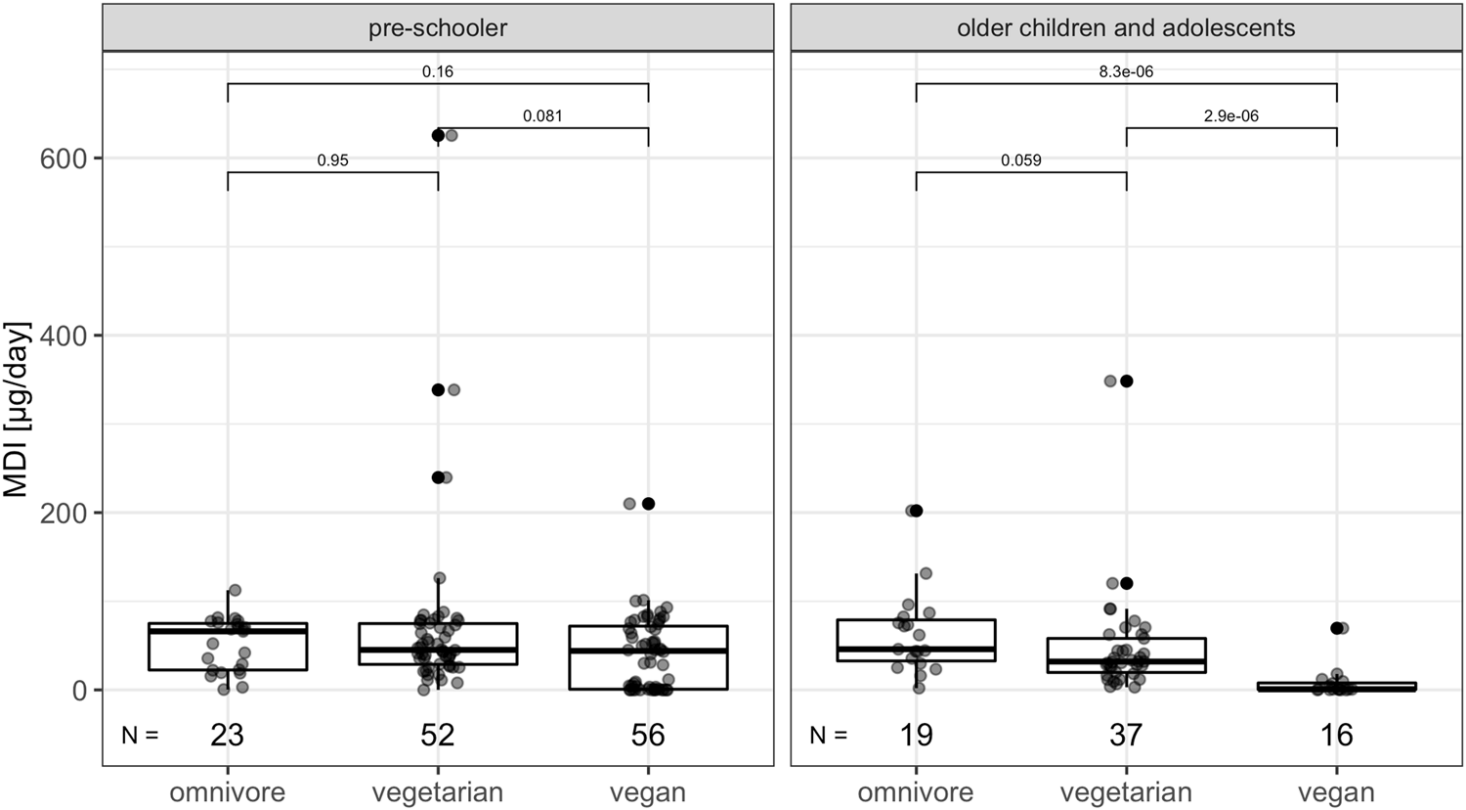
Daily dietary intake of iodine (ug/day). Pre-schoolers = children aged 0–6 years, older children and adolescents = children aged 6–18 years, MDI mean daily intake, line = median, box = interquartile range (IQR) e.g.,: Q1 = 25th percentile and Q3 = 75th percentile, whisker = Q1 − 1.5*IQR and Q3 + 1.5*IQR, *p* value calculated using Wilcoxon signed-rank tests.

According to our data 100% (*n* = 52) of OM children did not take any supplements with iodine content, followed by 83.7% (*n* = 77) VG and 78.2% (*n* = 61) VN children. We observed a significant difference in dosage of the supplementation between VN (26.54 µg/day ± 55.95)/VG (16.83 µg/day ± 41.82) group and OM (*p* < 0.001). There was a significant difference with moderate effect size in mean intake of iodine (without supplements) between VG (59.59 ± 82.18), with the highest intake and VN (33.86 µg/day ± 39.78) with the lowest intake (*p* < 0.001). When we examined the proportion of children who met the RDI as outlined in Table S2, only a small number of them demonstrated sufficient iodine intake solely through diet, without the use of any supplements. The findings are as follows: *n* = 2 OM (3.90%), *n* = 6 VG (6.70%) and *n* = 4 VN (5.2%) met the RDI through their dietary intake alone (*p* = 0.8). When we have performed the analysis with iodine supplements, the results were: *n* = 2 OM (3.90%), *n* = 14 VG (15.2%) and *n* = 12 VN (15.4%) (*p* = 0.09). For details on iodine intake, please see Tables 2–4 and Fig. 1.

**Table 2:** Cross-sectional comparison of iodine intake and selected blood markers between omnivore, vegetarian and vegan children aged 0–18 years.

Parameter	Control OM (*n*)	Min	Max	Mean (±SD)	Median	IQR	Diet (*n*)	Min	Max	Mean (±SD)	Median	IQR	p*	Adj. p**	Effect size CI 95%
Iodine intake															
MDI (µg/day)	42	0.40	202.10	56.58 (±38.34)	57.05	26.18–75.83	VG (89)	0.00	625.50	59.59 (±82.18)	40.80	26.30–70.20	0.24	0.49	0.26 (0.18–0.33)
							VN (72)	0.00	210.00	33.86 (±39.77)	11.80	0.70–65.12	<0.001	<0.001	0.31 (0.23–0.40)
Dose of supplement (µg/day)	52	0.00	0.00	0.00 (±0.00)	0.00	0.00–0.00	VG (92)	00.00	200.00	16.83 (±41.82)	0.00	0.00–0.00	<0.001	0.005	0.10 (0.13–0.46)
							VN (78)	0.00	200.00	26.54 (±55.95)	0.00	0.00–0.00	<0.001	<0.001	0.31 (0.23–0.40)
Markers of iodine status															
TSH [mUI/l]	51	0.77	6.70	2.62 (±1.42)	2.13	1.60–3.10	VG (89)	0.84	5.74	2.48 (±1.01)	2.28	1.76–3.16	0.91	1.00	0.01 (0.00–0.21)
							VN (74)	0.67	6.40	2.49 (±1.28)	2.20	1.58–3.04	0.72	1.00	0.03 (0.00–0.21)
fT4 [pmol/l]	51	11.21	18.16	14.99 (±1.74)	15.21	13.68–16.44	VG (89)	11.23	21.75	15.53 (±2.15)	15.46	14.12–16.68	0.23	0.23	0.10 (0.01–0.25)
							VN (74)	11.69	21.94	16.18 (±1.83)	15.96	15.22–17.10	0.00	0.00	0.28 (0.10–0.45)
fT3 [pmol/l]	51	4.92	8.53	6.97 (±0.79)	6.92	6.51–7.45	VG (89)	4.09	16.16	7.22 (±1.34)	7.16	6.55–7.65	0.28	0.28	0.09 (0.00–0.26)
							VN (73)	3.42	9.49	7.17 (±1.00)	7.24	6.70–7.83	0.08	0.16	0.16 (0.02–0.32)
TG [µg/l]	51	5.64	87.58	27.47 (±18.32)	22.18	13.86–40.54	VG (84)	0.39	104.7	20.15 (±20.66)	24.66	16.41–39.76	0.46	0.46	0.06 (0.00–0.24)
							VN (58)	1.99	345.50	35.33 (±44.50)	27.64	20.07–41.16	0.16	0.32	0.13 (0.01–0.34)
UIC [µg/l]	51	40.80	494.00	197.28 (±105.35)	180.00	118.50–253.50	VG (89)	15.70	787.00	177.95 (±155.88)	134.0	79.50–211.50	0.03	0.03	0.18 (0.04–0.33)
							VN (74)	5.99	991.80	162.97 (±164.51)	122.00	68.55–183.0	0.00	0.01	0.26 (0.09–0.42)

Values of blood markers were log transformed and adjustment was made for sex and age.

*MDI* mean daily intake, *TSH* thyroid-stimulating hormone, *fT4* free thyroxine, *fT3* free triiodothyronine, *TG* thyroglobulin, *UIC* spot urine iodine, *OM* omnivore, *VG* vegetarian, *VN* vegan, *n* number of subjects, *IQR* interquartile range, *SD* standard deviation, *CI* confidential interval.

*p value calculated using Wilcoxon signed-rank tests; ** adjusted *p* value calculated using linear regression.

**Table 3:** Cross-sectional comparison of iodine intake and selected blood markers between omnivore, vegetarian and vegan children aged 0–6 years.

Parameter	Control OM (*n*)	Min	Max	Mean (±SD)	Median	IQR	Diet (*n*)	Min	Max	Mean (±SD)	Median	IQR	p*	Adj. p**	Effect size CI 95%
Iodine intake															
MDI (µg/day)	23	0.40	112.5	51.53 ± 30.42	66.10	22.45–75.05	VG (52)	0.00	625.5	68.54 ± 95.47	45.15	28.77–74.88	0.24	0.24	0.01 (0.00–0.27)
							VN (56)	0.00	210.0	41.29 ± 41.33	44.05	0.78–72.00	0.00	0.00	0.16 (0.01–0.35)
Dose of supplement (µg/day)	28	0.00	0.00	0.00 ± 0.00	0.00	0.00–0.00	VG (54)	0.00	200.0	16.02 ± 43.14	0.00	0.00–0.00	0.00	0.00	0.23 (0.15–0.33)
							VN (60)	0.00	175.0	15.33 ± 40.25	0.00	0.00–0.00	0.00	0.00	0.23 (0.14–0.31)
Markers of iodine status															
TSH [mUI/l]	28	0.77	6.70	2.90 ± 1.72	2.46	1.45–4.09	VG (53)	1.23	5.74	2.64 ± 1.07	2.38	1.80–3.22	0.75	1.00	0.00 (0.01–0.29)
							VN (57)	0.67	5.09	2.48 ± 1.19	2.24	1.58–3.04	0.90	1.00	0.08 (0.00–0.29)
fT4 [pmol/l]	28	12.30	18.16	15.60 ± 1.62	15.64	14.21–16.89	VG (53)	11.23	21.75	16.02 ± 2.27	15.83	14.22–17.68	0.18	0.18	0.08 (0.00–0.27)
							VN (57)	13.35	21.94	16.46 ± 1.77	16.32	15.28–17.41	0.00	0.00	0.18 (0.01–0.38)
fT3 [pmol/l]	28	6.07	8.53	7.30 ± 0.70	7.29	6.79–7.87	VG (53)	6.19	16.16	7.61 ± 1.46	7.44	6.76–7.78	0.26	0.26	0.06 (0.00–0.27)
							VN (56)	6.02	9.49	7.47 ± 0.69	7.36	7.08–7.90	0.09	0.18	0.10 (0.01–0.33)
TG [µg/l]	28	8.35	87.58	35.47 ± 19.68	31.98	17.96–48.76	VG (49)	13.03	104.70	38.98 ± 21.03	34.70	23.54–44.46	0.50	0.50	0.17 (0.01–0.38)
							VN (44)	1.99	345.5	40.81 ± 49.70	32.47	23.40–43.01	0.12	0.24	0.00 (0.00–0.28)
UIC [µg/l]	28	40.80	494.0	190.84 ± 115.26	181.0	84.65–262.25	VG (53)	15.70	787.0	176.99 ± 174.76	115.0	68.40–211.00	0.03	0.03	0.17 (0.01–0.38)
							VN (57)	5.99	991.8	165.4 ± 184.3	101.0	52.8–187.0	0.01	0.01	0.21 (0.02–0.40)

Values of blood markers were log transformed and adjustment was made for sex and age.

*MDI* mean daily intake, *TSH* thyroid-stimulating hormone, *fT4* free thyroxine, *fT3* free triiodothyronine, *TG* thyroglobulin, *UIC* spot urine iodine, *OM* omnivore, *VG* vegetarian, *VN* vegan, *n* number of subjects, *IQR* interquartile range, *SD* standard deviation, *CI* confidential interval.

*p value calculated using Wilcoxon signed-rank tests; ** adjusted *p* value calculated using linear regression.

**Table 4:** Cross-sectional comparison of iodine intake and selected blood markers between omnivore, vegetarian and vegan children aged 6–18 years.

Parameter	Control OM (*n*)	Min	Max	Mean (±SD)	Median	IQR	Diet (*n*)	Min	Max	Mean (±SD)	Median	IQR	p*	Adj. p**	Effect size CI 95%
Iodine intake															
MDI (µg/day)	19	2.00	202.10	62.70 ± 46.32	45.90	32.70–79.05	VG (37)	3.00	348.30	47.02 ± 57.60	32.00	19.70–58.10	0.06	0.06	0.25 (0.02–0.51)
							VN (16)	0.00	69.60	7.84 ± 17.31	0.75	0.50–7.80	0.00	0.00	0.76 (0.59–0.85)
Dose of supplement (µg/day)	24	0.00	0.00	0.00 ± 0.00	0.00	0.00–0.00	VG (38)	0.00	150.00	17.97 ± 40.41	0.00	0.00–0.00	0.03	0.03	0.28 (0.17–0.39)
							VN (18)	0.00	200.00	63.89 ± 81.45	0.00	0.00–137.5	0.00	0.00	0.55 (0.37–0.74)
Markers of iodine status															
TSH [mUI/l]	23	0.86	4.35	2.26 ± 0.86	2.08	1.69–2.71	VG (36)	0.84	4.53	2.24 ± 0.88	2.08	1.72–2.52	0.94	1.00	0.01 (0.00–0.29)
							VN (17)	0.91	6.40	2.53 ± 1.61	1.90	1.58–2.48	0.81	1.00	0.04 (0.00–0.36)
fT4 [pmol/l]	23	11.21	16.97	14.25 ± 1.62	14.21	13.17–15.54	VG (36)	11.60	20.01	14.81 ± 1.76	14.74	13.63–15.96	0.31	0.31	0.13 (0.00–0.38)
							VN (17)	11.69	19.42	15.26 ± 1.76	15.28	14.26–16.22	0.08	0.15	0.28 (0.03–0.53)
fT3 [pmol/l]	23	4.92	7.75	6.56 ± 0.69	6.77	6.14–7.00	VG (36)	4.09	8.12	6.66 ± 0.89	6.81	6.11–7.25	0.51	0.51	0.09 (0.01–0.33)
							VN (17)	3.42	8.60	6.18 ± 1.22	6.25	5.29–6.87	0.20	0.41	0.20 (0.01–0.50)
TG [µg/l]	23	5.64	44.95	17.72 ± 9.91	15.65	10.62–23.00	VG (35)	0.39	54.30	17.79 ± 12.19	14.79	10.49–19.43	0.78	1.00	0.04 (0.00–0.30)
							VN (14)	4.98	37.15	18.13 ± 9.62	18.93	9.32–21.86	0.83	1.00	0.04 (0.01–0.39)
UIC [µg/l]	23	84.90	408.00	205.12 ± 93.81	177.00	156.5–238.50	VG (36)	40.10	700.00	179.37 ± 125.34	147.50	110.25–210.25	0.13	0.17	0.20 (0.01–0.44)
							VN (17)	50.80	316.30	154.72 ± 66.98	157.00	114.00–178.00	0.08	0.17	0.28 (0.03–0.55)

Values of blood markers were log transformed and adjustment was made for sex and age.

*MDI* mean daily intake, *TSH* thyroid-stimulating hormone, *fT4* free thyroxine, *fT3* free triiodothyronine, *TG* thyroglobulin, *UIC* spot urine iodine, *OM* omnivore, *VG* vegetarian, *VN* vegan, *n* number of subjects, *IQR* interquartile range, *SD* standard deviation, *CI* confidential interval.

*p value calculated using Wilcoxon signed-rank tests; ** adjusted *p* value calculated using linear regression.

### Biomarkers of thyroid and iodine status

**Fig. 2:**
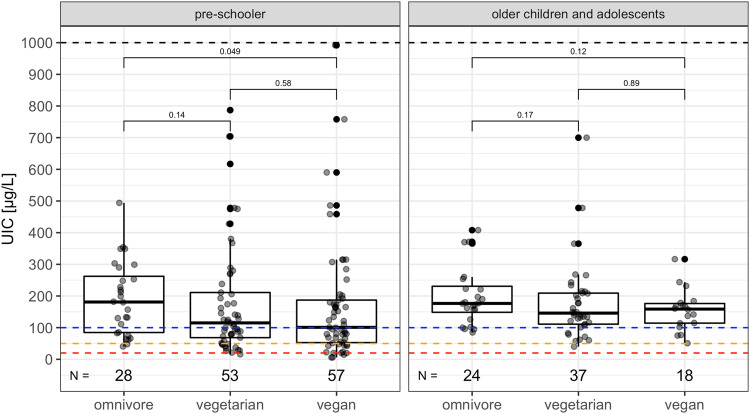
Iodine in spot urine (µg/l). UIC urinary iodine concentration, <100 µg/l = blue dotted line, <50 µg/l = yellow dotted line, <20 µg/l = red dotted line, pre-schoolers = children aged 0–6 years, older children and adolescents = children aged 6–18 years, line = median, box = interquartile range (IQR) e.g.,: Q1 = 25th percentile and Q3 = 75th percentile, whisker = Q1 − 1.5*IQR and Q3 + 1.5*IQR, *p* value calculated using Wilcoxon signed-rank tests.

The differences in evaluated biomarkers are reported in table (Tables 2–4). When comparing the whole sample (e.g., children aged 0.5–18.5 years old) we observed a statistically significant difference in UIC between the three groups, the highest values were observed in the OM group (197.28 ± 105.35 vs. VG: 177.95 ± 155.88 vs. VN: 162.97 ± 164.51 µg/l, *p* < 0.001). Lower values of UIC were observed in VN group compared to the VN group and particularly the OM group. The lowest (5.99 µg/l) but also the highest (991.80 µg/l) levels of UIC were measured in VN subjects. We identified *n* = 31 VN (41.9%), *n* = 31 VG (34.8%) and *n* = 10 OM (19.6%) children with iodine deficiency (e.g., UIC < 100 µg/l) (*p* = 0.06), out of which *n* = 8 VN, *n* = 11 VG and *n* = 2 OM with mild iodine deficiency (e.g., UIC < 50 µg/l) and *n* = 5 VN and *n* = 1 VG with severe iodine deficiency (e.g., UIC < 20 µg/l). In total the children with regular iodine supplement use had higher UIC; for children who did not take any supplements the median UIC was *n* = 75 VG 121 µg/l (75.2–208.50 µg/l) and *n* = 57 VN 101 µg/l (52.80–169.30 µg/l) compared to children who take the iodine supplements *n* = 14 VG 177.40 µg/l (149.85–268.73 µg/l) and *n* = 17 VN 231.90 µg/l (148.80–316.30 µg/l) (*p* < 0.001). The numbers of children with sufficient iodine concentration in morning spot urine were as follows: *n* = 43 (58.1%) in VN, *n* = 58 (65.2%) in VG, and *n* = 41 (80.4%) in OM (*p* = 0.03). The median UIC surpassed the World Health Organization (WHO) cut-off of 100 µg/l in all three groups. However, there is a noticeable trend of lower median UIC in the VN group, followed by the VG group, while the OM group exhibited the most favorable results. After stratifying the sample according to the age, a significant difference in urinary iodine concentration (UIC) persisted among the pre-schoolers, but it was not statistically significant among the older children and adolescents. This observation could be attributed to the reduction in sample size, potentially leading to a diminished impact on the results. There was also a significant difference in levels of fT4 with surprisingly highest levels in VN compared to OM (*p* < 0.001), this finding remains the same also after the division into two study groups only in pre-schoolers but is not significant in older children and adolescents. We found no significant differences in levels of TSH, fT3, or TG across the groups. To analyze the antibodies, we made the decision not to divide the group, as the criteria for determining positivity or negativity remained consistent across all age groups throughout the study duration. While there was no statistically significant difference in ATPOc positivity, the prevalence of AhTGc was significantly higher in the VG (18.2%)/VN (35.0%) groups compared to the OM group (2.1%) (*p* < 0.001), please refer to Table S3a, b. Concerning higher levels of ATPOc and AhTGc we retrospectively diagnosed *n* = 1 VN, *n* = 2 VG and *n* = 1 OM children with AIT, these outliers were omitted in data analysis, for data visualization please refer to Fig. 2.

## Discussion

The knowledge about the necessity of vitamin B12 supplementation is well-known among VN, and the prevalence of vitamin B12 deficiency is low as shown in a previous study [17] or Vechi Youth study [18]. On the other hand, we lack information about their iodine supplementation habits, nutritional intake, and iodine saturation. Therefore, we conducted this study to describe iodine saturation, intake, and supplement use in Czech children consuming plant-based diets and to assess whether these children are at possible risk of iodine deficiency, as reported in cross-sectional studies in adults. The study was designed as a cross-sectional study, and we examined a total of 222 children from November 2019 to July 2021 in our facility in the Czech Republic. Although the Czech Republic is considered a country that has solved iodine deficiency as a public health issue since 2004, we may have identified a new population at risk, namely children following a VN and VG diet. Unlike pregnant and lactating women, public health authorities have not paid attention to this group so far.

We observed that (1) the mean daily intake of iodine are significantly lower among VN compared to OM and VG children from the Czech Republic; (2) these differences were mirrored by a lower median UIC among VN children compared to OM children but all of them were >100 µg/l; (3) there were statistical significant differences in levels of fT4 between the groups, but no differences in TSH, fT3 and TG; (4) the VG/VN group exhibited a higher prevalence of AhTGc positivity, the difference in ATPOc was not statistically significant.

We observed the highest iodine intake among the VG group, lowest in the VN group, with intermediate intake levels in the OM control group. This is in partial agreement with other published studies where VN tend to have the lowest [19, 20] intake, although some studies show the highest intake of iodine compared to other groups [21, 22], possibly due to excessive seaweed consumption [20, 21, 23]. OM have the highest estimated average intake of iodine in most studies [10]. In one previous study among children, iodine intake was lowest in the VN group and highest in OM group, with intermediate intake levels among VG children [7]. VG children may have the highest intake because they tend to substitute meat with dairy and eggs that are the main source of iodine in diet when compared to meat in OM diet. Upon examining the proportion of children meeting the RDI, it was found that only a minority of them achieved sufficient iodine intake exclusively through their diet, without the use of any supplements. This discrepancy may be attributed to our oversight in accounting for the additional iodine content derived from table salt, as our calculations focused solely on the naturally occurring salt present in food. Another factor that could contribute to the disparity is the assumption that the breast milk of VN mothers is equivalent in iodine content to that of OM mothers, thus providing the necessary iodine. However, studies have revealed instances where VN mothers have inadequate iodine intake, resulting in lower iodine levels in their breast milk [24]. The dosage of supplement was the highest among VN followed by VG with no supplement use in OM group.

The overconsumption of algae is linked with the risk of exceeding the limits for iodine daily intake which may lead to thyroid dysfunction. This has been described in some case reports [13, 14], while we did not observe excess iodine consumption via algae in our cross-sectional study.

Even though iodine intake and status among consumers of plant-based diet are not sufficiently examined, there are several studies among adults to suggest that VN and VG diets are risk factors for developing iodine deficiency. These studies consistently describe a lowest UIC among VN, and the highest among the OM [8, 22, 25–29]. In addition, UIC among VG is higher than among VN yet lower than among OM people [8, 25–28]. Similarly, our results from a pediatric population indicate that UIC in VN children was the lowest followed by VG children and OM with the highest UIC. The children with regular use of iodine-containing supplements, tend to have higher UIC. Of note, none of our study participants had clinical signs of severe iodine deficiency; however, a clinical impact of moderate and mild iodine deficiency could not be properly assessed in the present cross-sectional study.

In our study we observe a higher prevalence of positive titers of AhTGc in the VG/VN group; this laboratory parameter is a marker of incipient autoimmune thyroid disease but also described in some studies as a marker of insufficient iodine intake [30–32]. The possible long-term results of prolonged mild and moderate iodine deficiency (e.g., mild cognitive impairment, school learning disabilities, autoimmune thyroid disease and others) [5] are yet to be determined using prospective cohort studies.

Diagnosing iodine deficiency has proven to be a complex task, even with an extensive range of laboratory tests, and has been a subject of scientific deliberation for a considerable period. In order to evaluate the iodine status of a specific population for epidemiological purposes, the recommended approach is to calculate the median UIC [5]. According to WHO, an ideal median UIC concentration should exceed 100 µg/l [5]. Although not used in our study, TSH levels obtained from new-born screening appear to be a valuable marker for assessing iodine sufficiency in new-borns [5]. Another population-level indicator of iodine deficiency is the prevalence of goiter, measured by palpation or by ultrasonography [5]. However, diagnosing iodine deficiency at an individual level remains challenging. Severe iodine deficiency may be characterized by elevated levels of fT3 compared to fT4, or subtle variations in TG levels [5]. Since the majority of iodine is excreted into urine, UIC can serve as a reasonably accurate marker to describe an individual’s iodine status, this method is widely use in studies comparing different diet groups (e.g., VG, VN, OM) [10]. It is important to note, however, that UIC exhibits significant intraindividual variability, with levels fluctuating considerably over the course of a few days and even throughout the day [33]. This variability can be mitigated by collecting the first morning urine sample, which tends to be the most concentrated. The gold standard for assessing UIC is a 24-h urinary collection, but this method is not widely applicable in children [5]. An alternative is to adjust UIC for urinary creatinine; however, this approach is not commonly recommended for children or individuals following VN/VG diets, as their creatinine intake may be affected by lower intake of meat and has never been validated in these groups [34]. Recent studies suggest that calculating the estimated 24-h UIC-to-creatinine ratio is the most suitable approach for children, as it considers their diverse body composition, including the percentage of water and water intake across different age groups [35, 36]. Again, this method is not widely applicable for assessing iodine deficiency in general practitioner outpatient settings. Therefore, the ideal, accessible, and most accurate tool for assessing iodine deficiency still requires further discussion and exploration.

A secondary outcome of our analysis, apart from the main hypotheses on differences in iodine intake and status, is our description of a higher number of *n* = 7 VN children with BMI values below the 3rd percentile. Recently published studies performed in a population of children consuming plant-based diets showed that VG/VN children are smaller and slimmer [37–39]. Some narrative reviews also discussing a possible relation between iodine deficiency and small growth [40].

Among other limitation of this study is that the sample was a convenience sample, which may introduce selection bias. Of note we observed a low recruitment response rate, particularly among adolescents and parents with alternative lifestyles. Furthermore, observation bias may have influenced our findings as the 3-day records were filled out by parents without any control. However, it is worth noting that previous research has shown that 3-day records can provide more accurate nutrient intake data compared to food frequency questionnaires, namely in VG and VN where FFQs tend to underestimate dietary intakes [41]. The high consistency of our results on differences in iodine intake and status across the groups with previous studies among adults indicates that selection or reporting bias may not have introduced differential measurement error, even when median UIC was used according to WHO recommendation for assessing iodine deficiency in the population. The non-significant differences in sex, weight, height is the relative balance between the study groups are the main strengths of our cross-sectional study. Moreover, the study contains detailed data about supplement use, and that the anthropometric measurements were taken using calibrated scales by a trained study nurse. Additionally, urine samples were collected as the first samples in the morning, and all laboratory analyses were performed immediately after the sample collection.

## Conclusion

We found that Czech VN and VG children are at the potential risk of iodine deficiency when they rely only on natural sources of iodine, namely when consuming a strictly plant-based diet (e.g., excluding meat, dairy and eggs). We observed more children with lower UIC, and trends to lower median UI (even when median UIC exceed the cut-off >100 µg/l in all three groups) in VN group than in VG or OM group. The positivity of AhTGc, an indicator of iodine deficiency, was more prevalent in VG/VN group. Only few children met the RDI via their diet. Regular use of iodine-containing supplements was associated with significantly higher levels of UIC. Apart from our focus on iodine intake and status, a higher number of VN children with lower BMI values, i.e., below the 3rd percentile, was observed. Our observations in a pediatric population concerning iodine saturation are in line with findings from other published studies predominantly carried out among adults [10].

The current study provides valuable insights into the iodine status of Czech children consuming plant-based diets and highlights the need for further research and evidence-based discussion regarding medical guidelines and recommendations for this population in European countries. In light of our findings, we recommend avoiding excessive consumption of algae, using iodized table salt when preparing homemade meals, and considering regular iodine supplementation within the daily recommended dosage range by the WHO, which includes 80 µg/day for children aged 1–3 years, 90 µg/day for children aged 4–6 years, 120 µg/day for children aged 7–9 years, and 150 µg/day for older children. Additionally, the possibility of a wide-reaching iodization of plant-based dairy alternatives may be a topic for further discussion.

### Supplementary information

Supplemental material

## Data Availability

The datasets generated during and analyzed during the current study are available from the corresponding author on reasonable request.
