# Supplementary material for: Prevalence of iodine deficiency among vegan compared to vegetarian and omnivore children in the Czech Republic: cross-sectional study
Source: Eur J Clin Nutr. 2023 Jul 24;77(11):1061–70. doi: 10.1038/s41430-023-01312-9 (PMC10630131; doi:10.1038/s41430-023-01312-9)
Supplement: Supplementary file 1 — Supplemental material [file 41430_2023_1312_MOESM1_ESM.docx]

**Figure S1.** STROBE Flowchart of a study design
VG = vegetarian, VN = vegan, OM = omnivore, n = number of participants

**
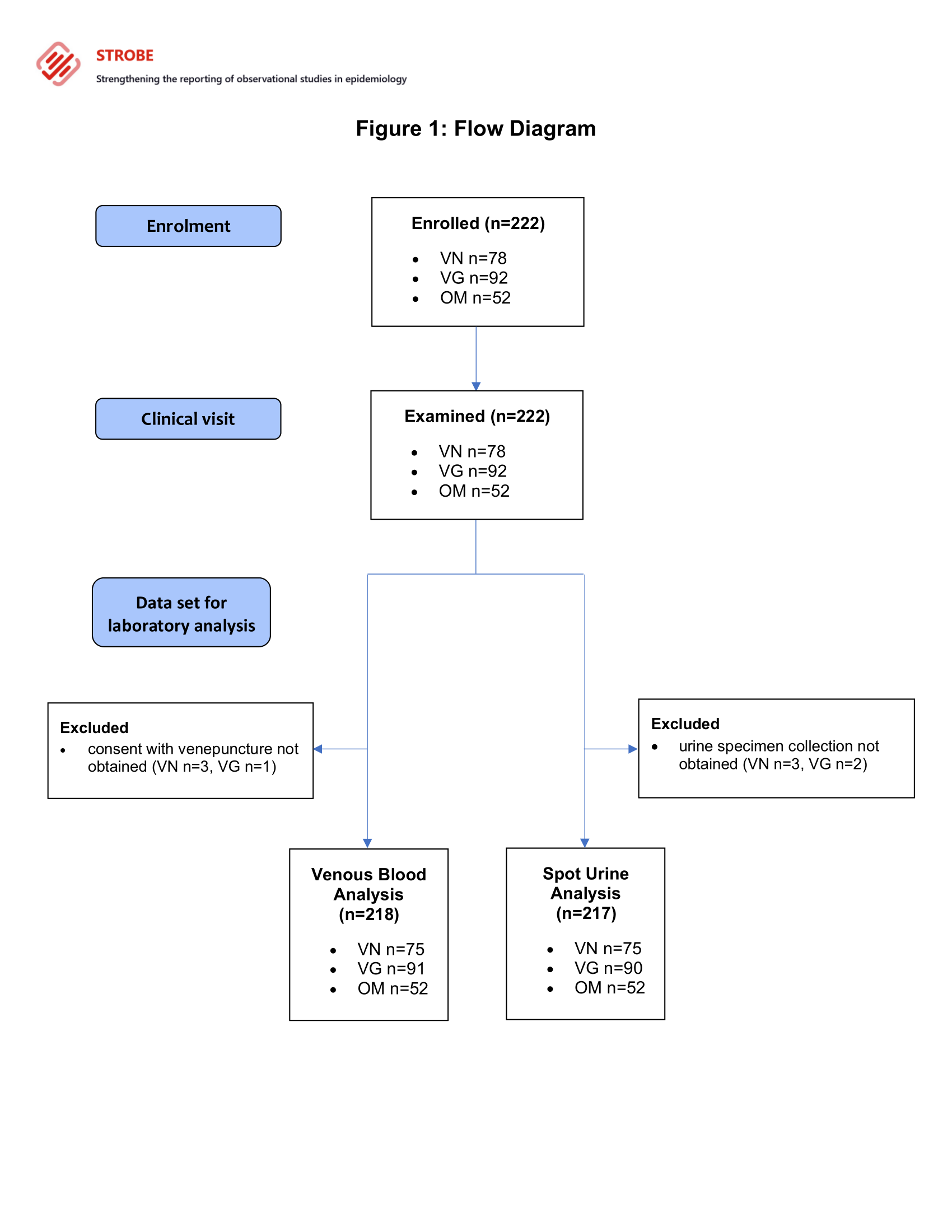
**

**Table S1.** Reference intervals of the laboratory parameters provided by manufacturer

|  | Lower Reference Limit | Upper Reference Limit |
| --- | --- | --- |
| **Thyroid-stimulating hormone (TSH)** |  |  |
| 6 months–2 years | 0.87 (mUI/L) | 6.15 (mUI/L) |
| 2 years–12 years | 0.67 (mUI/L) | 4.16 (mUI/L) |
| 12 years–18 years | 0.48 (mUI/L) | 4.17 (mUI/L) |
| **Thyroxine (fT4)** |  |  |
| 6 months–2 years | 12.1 (pmol/L) | 18.6 (pmol/L) |
| 2 years–12 years | 11.1 (pmol/L) | 18.1 (pmol/L) |
| 12 years–18 years | 10.7 (pmol/L) | 18.4 (pmol/L) |
| **Triiodothyronine (fT3)** |  |  |
| 6 months–2 years | 5.1 (pmol/L) | 8.0 (pmol/L) |
| 2 years–12 years | 5.1 (pmol/L) | 7.4 (pmol/L) |
| 12 years–18 years | 4.7 (pmol/L) | 7.2 (pmol/L) |
| **Thyroglobulin (TG)** | 3.5 (µg/L) | 77.0 (µg/L) |
| **Iodine in spot urine (UIC)** | > 100 (µg/L) |  |
| Mild iodopenia | 50 (µg/L) | 100 (µg/L) |
| Moderate iodopenia | 20 (µg/L) | 50 (µg/L) |
| Severe iodopenie | 0 (µg/L) | 20 (µg/L) |

TSH, fT4, fT3 analysed by chemiluminescence immunoassay automatically on the Siemens Atellica Solution system. TG by electrochemiluminiscent elecsys TG assay on the Roche Cobas e411 analyser. UIC by liquid chromatography. Reference values provided by the manufacturer

**Table S2.** Recommended daily intake of iodine according to age group (Assessment of iodine deficiency disorders and monitoring their elimination - WHO, UNICEF a ICCID 2007)

| Age group | RDI  µg/day |
| --- | --- |
| Children 0–59 months | 90 |
| Children 6–12 years | 120 |
| Adolescents | 150 |
| Pregnant and lactating women | 250 |

| Diet group | Negative n (%) | Positive n (%) |
| --- | --- | --- |
| OM | 33 (70.2 %) | 14 (29.8 %) |
| VG | 47 (61.8 %) | 29 (38.2 %) |
| VN | 24 (60.0 %) | 16 (40.0 %) |
| Total | 104 (63.8 %) | 59 (36.2%) |

**Table S3a.** Cross-sectional comparison of ATPOc positivity between omnivore, vegetarian and vegan children aged 0–18 years

| Diet group | Negative n (%) | Positive n (%) |
| --- | --- | --- |
| OM | 46 (97.9 %) | 1 (2.1 %) |
| VG | 63 (81.8 %) | 14 (18.2 %) |
| VN | 26 (65.0 %) | 14 (35.0 %) |
| Total | 135 (82.3 %) | 29 (17.7 %) |

**Table S3b.** Cross-sectional comparison of AhTGc positivity between omnivore, vegetarian and vegan children aged 0–18 years

ATPOc = anti-thyroid Peroxidase Antibody, AhTGc = anti-thyroglobulin antibodies, OM = omnivore, VG = vegetarian, VN = vegan, n= number of subjects, p value calculated using Wilcoxon signed-rank tests
